# Resistin: A Potential Biomarker for Periodontitis Influenced Diabetes Mellitus and Diabetes Induced Periodontitis

**DOI:** 10.1155/2014/930206

**Published:** 2014-02-12

**Authors:** Archana Devanoorkar, Rahul Kathariya, Nagappa Guttiganur, D. Gopalakrishnan, Paulami Bagchi

**Affiliations:** ^1^Department of Periodontics, A.M.E's Dental College and Hospital, Raichur, Karnataka 584103, India; ^2^Department of Periodontics and Oral Implantology, Dr. DY Patil Dental College and Hospital, Dr. DY Patil Vidyapeeth, Pune 411018, India; ^3^Department of Prosthodontics, Dr. DY Patil Dental School, Knowledge City, Lohegaon, Pune 412105, India

## Abstract

Biomarkers are highly specific and sensitive indicators of disease activity. Resistin is a recently discovered adipocytokine, having a potent biomarker quality. Initially resistin was thought to be produced by adipocytes alone; however, emerging evidence suggests that it is also produced in abundance by various cells of the immunoinflammatory system, indicating its role in various chronic inflammatory diseases. Data suggests that resistin plays a role in obesity, insulin resistance, cardiovascular diseases, and periodontitis. Resistin derived its name from the original observation that it induced insulin resistance (*resist-in: resist insulin*) in mice and is downregulated in mature murine adipocytes cultured in the presence of insulin sensitizing drugs like thiazolidinediones. It is well recognized that obesity, is associated with insulin resistance and diabetes. A three-way relationship has been established between diabetes, obesity and periodontitis. Recent evidence also suggests an association between obesity and increased risk for periodontitis. Our previous research showed incremental elevation of resistin with periodontal disease activity and a reduced level of resistin, after periodontal therapy. Thus resistin would be one of the molecular links connecting obesity, periodontitis, and diabetes and may serve as a marker that links periodontal disease with other systemic diseases. A Medline/PubMed search was carried out for keywords “Diabetes Mellitus,” “Periodontitis,” and “Resistin,” and all relevant research papers from 1990 in English were shortlisted and finalized based on their importance. This review provides an insight into the biological action of resistin and its possible role in periodontitis influenced diabetes mellitus and diabetes induced periodontitis.

## 1. Introduction

With the advancements in the technologies for the early detection, intervention, and prompt treatment of diseases, there is a growing scope for finding the most specific and sensitive biomarker.

“Biomarker is a characteristic that is objectively measured and evaluated as an indicator of normal biological or pathogenic processes, or as a pharmacologic response to a therapeutic intervention or other health care interventions” [1].

Biomarkers are potentially useful along the whole spectrum of the disease process. Before diagnosis, markers could be used for screening and risk assessment. During diagnosis, markers can determine staging, grading, and selection of initial therapy. Later, they can be used to monitor therapy, select additional therapy, or monitor recurrent diseases.

Resistin is one such newly recognised marker ensuring all qualities of an ideal biomarker. It is a cysteine-rich protein found in inflammatory zone [2]. It derived its name from the original observation that it induced insulin resistance in mice (*resist-in: resist insulin*) and it was downregulated in mature murine adipocytes cultured in the presence of insulin sensitizing drugs such as thiazolidinediones [3].

Studies have shown that levels of resistin are increased in various chronic inflammatory conditions such as rheumatoid arthritis, chronic kidney diseases, diabetic retinopathy, atherosclerosis, coronary heart diseases, and periodontitis [4].

Periodontitis results from the accumulation of bacterial plaque on the tooth surface. Plaque bacteria and its products, endotoxins, initiate the host immunoinflammatory response. This immunoinflammatory response of the host against microbes produces a bystander effect, that is, an attempt to ward off infection; it also results in local tissue destruction by producing various proinflammatory mediators [5].

Periodontitis is a common subclinical inflammatory condition characterized by increased production of pro-inflammatory mediators, such as prostaglandin E2 (PGE2), tumor necrosis factor- (TNF-) *α*, and interleukins IL-1 and IL-6. These mediators serve as important biomarkers of periodontitis [6]. Levels of these mediators increase in chronic periodontitis and coincide with the disease activity [7].

Chronic periodontitis being a low-grade infection is characterized by infiltration of the inflammatory cells within the periodontal tissues, which act as a source of production for resistin. Lipopolysaccharides (LPS) produced by periodontal pathogens are shown to induce the resistin gene in macrophages via cascade involving the production of proinflammatory mediators [8]. Pro-inflammatory mediators (PGE2, TNF-*α*, IL-1, and IL-6) produced during the periodontal pathogenesis in addition to local tissue destruction also exert certain systemic effects [9].

Periodontitis is considered to be the sixth complication of diabetes, and a bidirectional relationship has been established between diabetes and periodontitis, wherein one can influence the other [10]. Chronic subclinical inflammation has been shown to decrease the insulin sensitivity. Levels of resistin are found to be increased in periodontitis, and resistin plays an important role in inducing insulin resistance, thus increasing the risk for type II diabetes [6]. Obesity, which is another important risk factor for type II diabetes, has also been linked positively to periodontitis [6].

Obesity is characterized by increase in the adipose tissue, which is an important source for various pro-inflammatory cytokines such as resistin, TNF-*α*, IL, visfatin, and adiponectin. Obesity is characterized by the presence of chronic subclinical inflammation with increased concentration of the abovementioned proinflammatory cytokines [11].

Obesity, diabetes, and chronic periodontitis are related to each other wherein obese subjects are at increased risk for diabetes as well as chronic periodontitis, thereby exhibiting a triangular relationship among the above three conditions, and resistin may act as a connecting molecular link between these conditions [11].

Clinicians over the years have been working on different biological markers to establish a link between periodontal diseases and various systemic conditions and to determine which of those patients are at risk to develop the latter. Substances such as C-reactive protein (CRP), aspartate amino transferase (AST), tumor necrosis factor-alpha (TNF-*α*), and prostaglandin (PG) E2 have been extensively assayed for periodontitis leading to other subclinical infection.

Our previous studies evaluated many biomarkers in periodontitis leading to chronic systemic infections. Biomarkers like CRP [12], pentraxin-3 [13, 14], visfatin [15, 16], cystatin [17], and resistin [18] were evaluated for their presence in periodontitis relating to chronic kidney diseases [19] and diabetes [20].

Resistin could be one such marker that could provide a link among the periodontitis-obesity-diabetes mellitus triad. A Medline/PubMed search was carried out for keywords “Diabetes Mellitus,” “Periodontitis,” and “Resistin,” and all relevant research papers from 1990 in English were shortlisted and finalized based on their importance. This review is intended to give an insight into the biological action of resistin and its role in periodontitis influenced diabetes mellitus and diabetes induced periodontitis.

## 2. Biomarkers in the Diagnosis, Treatment, and Prognosis of a Disease

Biomarkers span a broad sector of human health care and have been around since the understanding of human biology and diseases began to evolve. Genetics, genomics, proteomics, and modern technologies allow us to measure more markers than before. In addition, we achieve a greater understanding of disease pathways, targets of interventions, and the pharmacologic consequences of medicines. The biomarker is either produced by the diseased organ (e.g., tumour) or by the body in response to disease. Biomarkers are potentially useful along the whole spectrum of the disease process.

## 3. History of Resistin

Resistin is a recently discovered adipocyte-secreted polypeptide that has been implicated in the development of insulin resistance. Resistin was first described in 2001 during a search for genes that induced adipocyte differentiation, and it was downregulated in mature adipocytes during exposure to TZD. This led to the discovery of a protein that the investigators named as resistin (for resistance to insulin) [21].

Resistin is a member of a family of tissue-specific signaling molecules, called resistin-like molecules. The resistin gene is expressed in white adipose tissue in mice and rats, and it is also present in immunocompetent cells [3, 21]. It belongs to the “found in inflammatory zones” family (FIZZ) (now also known as RELMs, i.e., resistin-like molecules). The first member in this family to be discovered, FIZZ1 (also known as RELM-*α*), is a protein that is found in above normal levels in bronchoalveolar fluid of mice with experimentally induced asthma [22]. FIZZ2 (RELM-*β*) was discovered in the proliferating epithelium of intestinal crypt [22]. Resistin (FIZZ3) has been found in adipocytes, macrophages, and other cell types. In rodents, a fourth FIZZ protein, RELM-*γ*, has been identified in WAT and haematopoietic tissues [22, 23]. Resistin is related to resistin-like molecules *α*, *β*, and *γ* in structure and function.

Resistin is mainly secreted by adipocytes in mice [24] and by mononuclear cells [25] in humans. Mouse resistin, a cysteine-rich protein primarily secreted from mature adipocytes, is involved in insulin resistance and type 2 diabetes. Human resistin, however, is mainly secreted by immune mononuclear cells, and it competes with lipopolysaccharide for the binding to Toll-like receptor 4, which could mediate some of the well-known proinflammatory effects of resistin in humans [26].

Resistin is produced by white and brown adipose tissues but has also been identified in several other tissues, including the hypothalamus, pituitary and adrenal glands, pancreas, gastrointestinal tract, myocytes, spleen, white blood cells, and plasma. It antagonizes insulin action, and it is downregulated by rosiglitazone and peroxisome proliferator-activated receptor agonists [22, 27].

### 3.1. Resistin (FIZZ3)

Found in inflammatory zone, adipocyte secreted factor (ADSF) is a cysteine-rich, 108-amino acid peptide hormone with a molecular weight of 12.5 kDa. Recent reports have now shown that human resistin has 108 amino acids [11], while rat and mouse resistin have 114 amino acids. Cysteine is the most common amino acid in resistin, where it forms approximately 12% of its amino acid sequence.

### 3.2. RELM-*α*

RELM-*α* has been identified in both rat and mouse tissues. In rat, it is found on chromosome 11 and located at II q21. However, it is located at chromosome 16; 16Al in mouse. RELM-*α* messenger RNA (mRNA) is expressed in white adipose tissue of the heart, lung, and tongue but not expressed in 3T3-Ll adipocytes nor in pre-adipocytes [3]. RELM-*α* is also present in the inflammatory zone of mice with allergic pulmonary inflammation [11].

### 3.3. RELM-*β* (FIZZ2)

Studies using RT-PCR analysis have demonstrated that RELM-*β* mRNA is found only in the undifferentiated, proliferating colonic epithelial cells of mouse. It is absent in adipose tissue of mouse. RELM-*β* was first located within the proliferating cells of the colonic epithelium and disappears as soon as the cell becomes mature. Moreover, high levels of RELM-*β* can be detected in the stool of humans [2].

### 3.4. RELM-*γ*

It is the most recently discovered member of the RELM family and was first observed in nasal respiratory epithelium of rats exposed to cigarette smoke. The highest expression of RELM-*γ* was found in haematopoietic tissues, indicating a cytokine-like function for RELM-*γ*. RELM-*γ* is also expressed in white adipose tissue of rat and is similar to RELM-*α* [23].

## 4. Cells Producing Resistin

Initially it was thought that resistin is mainly produced by adipocytes. However, recent studies have shown that very little resistin is produced by adipocytes, whereas large amount of resistin is produced from cells of the immunoinflammatory system like PMNs, monocytes, and macrophages [11]. Resistin is a member of a family of tissue-specific signaling molecules called as resistin-like molecules.

## 5. Characteristics of Resistin and Other Adipokines (Table 1)

Adipocytokines are bioactive mediators released from the adipose tissues including adipocytes and other cells present within fat tissues. These include several novel and highly active molecules released abundantly by adipocytes like leptin, resistin, adiponectin, and visfatin, as well as some more classical cytokines released possibly by inflammatory cells infiltrating fat, like TNF-*α*, IL-6, MCP-5061 (CCL-2), and IL-1 [30].

## 6. Role of Resistin in Systemic Diseases

### 6.1. Involvement of Resistin in Insulin Resistance

The effects of resistin on the expression and localization of GLUT (glucose transporter) and on the development of insulin resistance have been extensively studied [31]. In mice, neutralization of resistin by antibody improved glucose and insulin action in diet-induced obesity [11]. Two independent groups reported that administration of recombinant resistin impaired glucose tolerance and insulin action [11, 22]. The role of resistin in insulin resistance was also investigated in mice, engineered to knockdown or overexpress resistin. Knockdown of resistin can completely reverse the hepatic insulin resistance in diet-induced insulin resistant mice [30]. Overexpression of resistin in circulation by adenovirus-mediated gene expression led to glucose intolerance, hyperinsulinemia, and hypertriglyceridemia associated with impaired insulin signaling in skeletal muscle, liver, and adipose tissue [32]. Although first identified in mice, it is now being found in humans [33].

It is generally accepted that chronic exposure to resistin adversely affects the entry of glucose into cells, thus counteracting the effect of insulin. Overexpression of receptor tyrosine kinase-like orphan receptor- (ROR-) 1 induces the expression of GLUT1 and GLUT4 in 3T3-L1 preadipocytes, and this effect can be reversed by resistin. On the other hand, overexpression of ROR1 blocks adipogenesis, and resistin is able to enhance this differentiation process by inhibiting the activity of ROR1 [34].

Mouse receptor tyrosine kinase-like orphan receptor- (ROR-) 1 could mediate some of the described functions of resistin in 3T3-L1 adipogenesis and glucose uptake. This study demonstrated the interaction of mouse resistin with specific domains of the extracellular region of the ROR1 receptor. This interaction results in the inhibition of ROR1 phosphorylation, modulates ERK1/2 phosphorylation, and regulates suppressor of cytokine signaling 3, glucose transporter 4, and glucose transporter 1 expression [35].

From the observations of the above study it was concluded that mouse resistin is a potential inhibitory ligand for the receptor ROR1 and that ROR1 plays an important role in adipogenesis and glucose homeostasis in 3T3-L1 cells. These data could explain the important relationship of the mechanism of action of resistin in adipogenesis and in the development of insulin resistance. In a study by Zhou et al., the effect of resistin on glucose tolerance in adult human hepatocytes (L-02 cells) is determined. To understand its molecular mechanism, mRNA levels of key genes in glucose metabolism and insulin signaling pathway were analyzed, and the results demonstrated resistin-stimulated expression of glucose-6-phosphatase (G6Pase), sterol regulatory element-binding protein 1c (SREBP1c) and suppressor of cytokine signaling-3 (SOCS-3), repressed expression of peroxisome proliferator-activated receptor *γ* (PPAR*γ*), and insulin receptor substrate-2 (IRS-2). Resistin lowered mRNA levels of IRS-2 while stimulating SOCS-3 expression, which suggests it impairs glucose tolerance by blocking the insulin signal transduction pathway [36].

All of these findings clearly suggest a pivotal role of resistin in insulin resistance and type 2 diabetes, although the receptor and relevant intracellular signaling pathway of resistin have not yet been completely understood.

Nonetheless, Utzschneider et al. did not find any effect of resistin on insulin sensitivity or the metabolic syndrome in humans. The authors intended to determine the relationship between resistin and insulin sensitivity, body fat distribution and the metabolic syndrome in humans. When subjects were divided into categories based on BMI (< or ≥27.5 kg/m^2^) and SI (< or ≥7 × 10^−5^ min^−1^ [pmol/L]^−1^), resistin levels did not differ between the lean, insulin sensitive (*n* = 53, 5.36 ± 0.3 ng/mL), lean, insulin-resistant (*n* = 67, 5.70 ± 0.4 ng/mL), and obese, insulin-resistant groups (*n* = 48, 5.94 ± 0.4 ng/mL). The researchers concluded that, in contrast to other adipokines, resistin is only weakly associated with body fat and is unlikely to be a major mediator of insulin resistance or the metabolic syndrome in humans [37].

### 6.2. Obesity and Systemic Inflammation

For many years, adipose tissue was considered as an inert organ that stored triglycerides. It is now clear that adipose tissue is a complex and metabolically active endocrine organ that secretes numerous immunomodulatory factors and plays a major role in regulating metabolic and vascular biology. Adipose cells, which include adipocytes, preadipocytes, and macrophages, secrete more than 50 bioactive molecules, known collectively as adipokines [35]. Adipokines play a number of different roles, such as hormone-like proteins (e.g., leptin and adiponectin, resistin), classical cytokines (e.g., tumor necrosis factor-*α*, interleukin-6), proteins involved in vascular hemostasis (e.g., plasminogen activator inhibitor-1, tissue factor), regulators of blood pressure (angiotensinogen), promoters of angiogenesis (e.g., vascular endothelial growth factor), and acute phase respondents (e.g., C-reactive peptide) which play an important role in atherogenesis and increasing insulin resistance [38].

Around 90% of the individuals who develop type 2 diabetes have a body mass index higher than 23.0 kg/m^2^. The relative risk for an obese person to develop type 2 diabetes is 10-fold for women and 11.2-fold for men [32]. Diabetes risk is significantly increased by early weight gain, with increased abdominal obesity, or in patients with a history of maternal gestational diabetes [39, 40]. Insulin resistance is considered to be the major underlying abnormality.

### 6.3. Resistin and Inflammatory Diseases

Although resistin was firstly postulated to contribute to insulin resistance, more and more evidence indicated that it may also be involved in inflammatory process. Some proinflammatory agents, such as tumor necrosis factor-*α* (TNF-*α*), interleukin-6 (IL-6), and lipopolysaccharide (LPS), can regulate resistin gene expression. Resistin mRNA was strongly increased by TNF-*α* in human peripheral blood mononuclear cells (PBMC) [41].

Recent studies have shown the regulation of proinflammatory cytokine expression by resistin. Resistin strongly upregulated IL-6 and TNF-*α* in human PBMC via NF-*κ*B pathway [42].

Addition of recombinant human resistin protein to macrophages from both mice and humans resulted in enhanced secretion of proinflammatory cytokines, TNF-*α*, and IL-12 [42]. LPS was reported to induce resistin gene expression in primary human macrophages via cascade involving the secretion of inflammatory cytokines [8].

Another evidence linking resistin to inflammation is that plasma resistin levels were found associated with many inflammatory markers in some pathophysiological conditions. Resistin level was also positively associated with levels of inflammatory markers, including soluble TNF-*α* receptor-2, IL-6, and lipoprotein-associated phospholipase A_2_ in atherosclerosis patients [43].

### 6.4. Resistin and Atherosclerosis

Verma et al. found that resistin promoted endothelial cell activation by promoting endothelin-1 release, partly by inducing endothelin-1 promoter activity. Furthermore, resistin upregulated vascular cell adhesion molecule-1 (VCAM-1) and monocyte chemotactic protein-1 (MCP-1) and downregulated TNF-receptor-associated factor-3, an inhibitor of CD40 ligand signaling which can induce MCP-1 production [44].

In a study done to determine the levels of serum resistin between patients with stable and unstable angina it was found that plasma concentrations of resistin were significantly increased in unstable angina group in comparison with stable angina group and control groups. However, no differences in resistin levels were found between patients with stable angina group and controls. It was also found that plasma resistin positively correlated with leukocyte counts, high sensitive C-reactive protein, and endothelin-1 after adjustment for age, sex, and BMI. It was concluded that resistin may be involved in the development of CAD by influencing systemic inflammation and endothelial activation [45, 46].

Another study done to determine the effects of resistin on CVD indicated that resistin aggravates atherosclerosis by stimulating monocytes, endothelial cells, and vascular smooth muscle cells to induce vascular inflammation. These findings provide an insight into the causal relationship between resistin and atherosclerosis [47].

### 6.5. Resistin and Chronic Kidney Diseases

A study was done to determine the levels of resistin, adiponectin, and other inflammatory markers in subjects with chronic kidney diseases with those of control subjects, and it was found that subjects with chronic kidney diseases have increased levels of resistin and TNF-*α*. Authors concluded that the increased levels of resistin and TNF-*α* in subjects with CKD suggest that these mediators may play a role in the subclinical inflammation associated with CKD [48].

### 6.6. Resistin and Rheumatoid Arthritis

In human studies, synovial fluid from patients with rheumatoid arthritis (RA) showed significantly higher level of resistin compared with control samples. Moreover, resistin level in RA synovial fluid positively correlated with synovial leukocyte count and IL-6 level [41]. Thus, the role of resistin in RA is apparent, but the underlying mechanism needs further investigations.

### 6.7. Resistin and Chronic Periodontitis

Chronic periodontitis being a disease of multifactorial etiology is characterized by stimulation of host immune-inflammatory system in response to microbial deposits and their endotoxins produced. This results in a chronic low grade subclinical inflammation. Host immune-inflammatory system, in an attempt to ward off the infections, causes the infiltration of periodontal tissues by various immune-inflammatory cells such as PMNs and monocytes, macrophages [49, 50]. These cells and the cytokines produced such as TNF-*α*, CRP, interleukins, prostaglandins, and resistin not only result in the local tissue destruction characterized by periodontal attachment loss and alveolar bone loss but also exert certain distant systemic effects such as increased risk for atherosclerosis, PTLBW, and increased insulin resistance [50].

Previous studies have reported that there is increased level of proinflammatory mediators such as TNF-*α*, CRP, and PGE2 their increased levels subsequently increase systemic morbidity. The role of resistin in insulin resistance and various chronic diseases is well established as mentioned in the above studies [4, 51].

Furugen et al. in their study investigated the levels of adipokines such as resistin, adiponectin, TNF-*α*, and IL-6 in chronic periodontitis subjects in elderly Japanese individuals. The authors reported that serum resistin levels were higher in chronic periodontitis patients compared with the healthy counterparts [9].

In a Hisayama study of middle-aged women with moderate to severe chronic periodontitis done to determine the levels of circulating serum resistin and adiponectin, it was observed that patients with chronic periodontitis had increased serum resistin levels. It was concluded from the study that the increased levels of serum resistin in middle-aged women might affect the systemic health [52].

In our previous study, it was observed that there were increased levels of serum resistin in periodontitis subjects compared with the control group, although this difference was not statistically significant. Additional finding of the study was that the levels of serum resistin decreased in subjects of the study group following the nonsurgical periodontal therapy [18].

In a recent study done to determine the resistin levels in GCF of patients with chronic periodontitis and healthy controls, it was observed that there were significantly increased levels of resistin in chronic periodontitis compared with the healthy controls [53].

Zimmermann et al. determined serum and GCF levels of adipokines in obese and normal weight subjects, with and without chronic periodontitis. It was observed that the resistin levels were higher while adiponectin levels were lower in periodontitis group compared to the nonperiodontitis groups. The authors concluded that periodontitis mainly influenced the circulating levels of resistin and adiponectin [54].

## 7. Conclusion

At present there are several biomarkers, studied in relation to periodontitis and diabetes. However, there are very few studies in the field of periodontics addressing the relation between chronic periodontitis and resistin, which acts as a biomarker “the connecting link between periodontitis, obesity, and diabetes.” The currently available literature suggests that the levels of resistin are increased in the patients with chronic periodontitis compared to the clinically healthy controls. Resistin induces insulin resistance, and large amount of resistin is secreted by adipocytes. Increased resistin levels in periodontitis may thus be considered to pose a risk for diabetes by decreasing the insulin sensitivity. Thus, periodontitis might lead to development of type II diabetes or diabetes might influence the occurrence or progression of periodontitis. Identification of resistin may lead to a specific and sensitive diagnosis. To the best of our knowledge there is only one study [18] that has assessed the effect of periodontal therapy on resistin levels, results of which showed that there was decrease in the serum resistin levels following nonsurgical periodontal therapy in chronic periodontitis patients. This indicates that increased systemic burden of proinflammatory cytokines can be reduced by periodontal therapy, thus improving the patient's overall systemic condition. Thus, based on the results of the present study, it can be observed that resistin can serve as one of the potential biomarkers for periodontitis with other systemic diseases such as diabetes and cardiovascular diseases. However, further long-term and interventional studies with larger sample sizes are warranted, to give a direct cause-effect relationship between resistin and chronic periodontitis and to determine the exact molecular mechanism involved in increased insulin resistance.

## Figures and Tables

**Table 1:** Major effects of key adipocytokines on the immune and vascular systems [28].

Adipocytokine	Immune system effects	Vascular effects [29]
Leptin	Inflammatory increase in T cell activation and cytokine release proliferation promotes Th1 response increases NK cell activation Increases macrophage activation and cytokine release (TNF-a/IL-6 etc.) Activates neutrophils and increases their chemotaxis and oxidative burst Prevents inflammatory damage in conditions of overt immune system stimulation	Induces endothelial dysfunction Increases blood pressure Atherosclerosis Increases ICAM and VCAM Plasma levels related to hard clinical endpoints but acutely releases NO from endothelium

Adiponectin	Anti-inflammatory Decreases T cell activation and proliferation Inhibits NF-*κ*B dependent cytokine release and adhesion molecule expression (including TNF-a/IL-6) Increases IL-10 Inhibits phagocytosis and oxidative burst	Vasculoprotective Prevents atherosclerosis Is decreased in hypertension Correlated with HDL and inversely with LDL Plasma levels not related to hard endpoints

Resistin	Proinflammatory Activates NF-*κ*B dependent cytokine release and adhesion molecule expression (including TNF-a/IL-6)	Pathogenic Impairs bradykinin dependent vasorelaxations (NO and EDHF) No effect on acetylcholine dependent vasorelaxations (NO) VEGF and MMP up regulation
